# Drought-attributable mortality in Somalia, 2015–2024: a retrospective and modelling study

**DOI:** 10.1016/j.lanafr.2026.100072

**Published:** 2026-10

**Authors:** Yamna Ouchtar, Dahir Abdi Ali, Yahye Abukar Ahmed, Mohamed Hussein Adam, Najib Isse Dirie, Abdihamid Warsame, Mohamed Yusuf, Chris Grundy, Oliver John Watson, Francesco Checchi

**Affiliations:** a Faculty of Epidemiology and Population Health, London School of Hygiene and Tropical Medicine, London, United Kingdom; b Faculty of Economics, SIMAD University, Mogadishu, Somalia; c Faculty of Computing, SIMAD University, Mogadishu, Somalia; d Faculty of Medicine and Health Science, SIMAD University, Mogadishu, Somalia; e Mogadishu Institute of Health, Mogadishu, Somalia; f World Health Organization, WHO, Mogadishu, Somalia; g Applied Health Analytics for Delivery and Innovation, Nairobi, Kenya; h Faculty of Medicine, Imperial College, London, United Kingdom

**Keywords:** Excess mortality, Drought, Somalia, Humanitarian crisis, Child mortality, Statistical modelling

## Abstract

**Background:**

Over the past 17 years Somalia and the Horn of Africa have experienced four droughts. We aimed to retrospectively estimate excess population mortality across Somalia during a period encompassing the last two droughts.

**Methods:**

We estimated all-age and under 5 years old excess mortality by district and month by (i) reanalysing household mortality surveys; (ii) reconstructing population denominators from census records and displacement data; (iii) assembling predictors of mortality; (iv) fitting a model to predict death rates and tolls; and (v) subtracting counterfactual mortality levels, corresponding to non-drought periods.

**Findings:**

Assuming pre-2017 levels as counterfactual, we estimated 71,100 excess deaths across Somalia during the 2022–2024 period, 64,500 (91%) of which were among children under 5 years. During the 2017–2019 period, an estimated 79,600 excess deaths occurred, 49,000 (61%) among children under 5 years. An alternative counterfactual based on 2020 trends yielded a higher number of excess deaths. Regional disparities were marked: north-eastern regions had relatively low mortality despite high food insecurity, while south-central areas (including Banadir, Bay, and Lower Shabelle) bore the majority of excess deaths.

**Interpretation:**

The mortality impact of the latest drought crisis (2022–2024) in Somalia appears comparable to that witnessed during the 2017–2019 period. The combination of intensifying droughts attributable to climate change in the Horn of Africa, substantially declined global humanitarian assistance budgets, and steep population growth threaten to cause persistent and potentially worsened health impacts over the coming decades, including a large number of lives lost.

**Funding:**

This study was funded by the United Nations Children's Fund (UNICEF) East Africa office, award number 2022/1291432-0.

**Research in context**

**Evidence before this study**

We searched PubMed, Scopus, and Web of Science for articles published between Jan 1, 2000, and Dec 31, 2024, using combinations of the search terms “Somalia”, “mortality”, “excess mortality”, “drought” and “food security”. We also searched grey literature sources, including reports from the UNICEF, World Health Organization, and Food and Agriculture Organization.

Existing data consistently highlight that accurate estimates of mortality in crisis situations are essential for setting humanitarian priorities and ensuring accountability. However, for the drought between 2017 and 2019 in Somalia, published work has largely focused on food security and malnutrition indicators or has presented localised mortality results from small-area assessments and rapid surveys. National and temporal estimates of excess mortality, particularly those specific to age and subnational levels, have been rare, methodologically heterogeneous, and rarely linked to concomitant factors such as displacement, conflict, market shocks, and rainfall anomalies.

**Added value of this study**

This study provides a rigorous and transparent estimate of excess mortality associated with the 2017–2019 drought in Somalia, quantifying tens of thousands of additional deaths and demonstrating a disproportionate burden among children under five. We aggregate and harmonise several regularly available data streams (humanitarian and government assessments, displacement flows, conflict events, climate and market indicators, health and nutrition surveillance) into a coherent mortality estimation framework. This framework is fully reproducible and adaptable to other crisis contexts where no reference civil registry exists, and emphasises transparency of assumptions, characterisation of uncertainties, and sensitivity analyses relevant to operational decision-making.

**Implications of all the available evidence**

Our findings show that significant mortality rates can occur during periods of severe drought and insecurity, and that young children account for a significantly higher proportion of deaths. In terms of policy and operations, this implies three priorities: (1) investing in granular and up-to-date data systems, including displacement tracking, nutrition and morbidity monitoring, and market and climate monitoring, in order to infer mortality risk before it is reflected in crude mortality rates; (2) integrate early warning triggers that incorporate these signals in order to adapt aid earlier, explicitly targeting the reduction of under-five mortality (e.g., integrated management of childhood illnesses, measles and cholera prevention, ready-to-use therapeutic food supply chains, and WASH); and (3) adopt routine and reproducible mortality estimation as a standard accountability indicator, alongside IPC/food security analyses. Prospective application of this framework can refine geographic targeting, protect children, and provide clearer accountability for the human consequences of drought.

## Introduction

In Somalia, a confluence of climate change and three decades of armed conflict has kept much of the population in a chronic cycle of vulnerability. Since 2008, four recognised drought periods have affected the country. The 2010–2012 drought occurred so soon after the previous instance (2007–2008) that many affected communities were unable to replenish their depleted coping mechanisms. This triggered a severe famine, particularly affecting south-central regions, and exacerbated by a delayed, politically constrained response.^1^ A study commissioned by the United Nations and employing an early version of the current analysis attributed around 256,000 deaths to acute food insecurity during this period, half of which were children under the age of 5 years.^2^ This crisis also triggered large-scale displacement and epidemics of measles, cholera, and pertussis.

Somalia has two annual rainy seasons: the *gu* rains (April to June) and *deyr* rains (October–December). Since 2012, few seasons have followed typical patterns, alternating lower-than-expected rainfall and exceptional floods. By 2016, successive poor harvests and resurgent insecurity, compounded by the return of refugees from neighbouring countries, had pushed many Somalis back into acute food insecurity.^3^^,^^4^ The 2017–2019 drought crisis was again characterised by food insecurity, mass displacement towards urban centres, and epidemics, although the response was more proactive than in previous crisis. After failed rains in 2022, the Integrated Phase Classification (IPC) system projected that 6.6 million Somalis, or 39% of the population, would be in IPC food insecurity phases 3–5 during April to June 2023.^5^ This new drought (2022–2023), the fourth to hit Somalia since 2007, followed livelihood losses during the COVID-19 pandemic and the pressure of rising food prices. Droughts may cause excess mortality through complex causal pathways, involving upstream (climate, food insecurity) and downstream (forced displacement, malnutrition, a heightened burden of infectious disease) factors. These effects are further shaped by the availability of coping strategies and the coverage and effectiveness of humanitarian assistance. Evidence on excess mortality can inform humanitarian responses, facilitate the mobilisation of resources, and highlight the human impact of droughts, including the human-made drivers, such as insecurity and climate change. However, vital registration systems in many drought-affected countries, including Somalia, remain inadequate, potentially obscuring the public health impact of resulting crises.

In earlier work, we estimated excess mortality during the 2017–2019 drought.^6^ Here, we present an analysis of mortality over the decade 2015 to June 2024, using a larger dataset and updated methods. We deliberately include non-drought periods to construct locally appropriate counterfactual mortality levels (i.e., expected mortality in the absence of crises).

## Methods

### Study population and period

The analysis covered the population of Somalia, including Somaliland and Puntland but excluding refugees, from January 2015 to June 2024. We stratified estimates by month and district as per United Nations administrative boundaries: note that Banadir region, where the capital Mogadishu is located, is considered a single district. The study period (2015–2024) was used to reconstruct population denominators and baseline mortality trends, while excess mortality estimates focused on specific drought periods (2017–2019 and 2022–2024), chosen based on drought timing.

### Study design

We refined a previously established small-area statistical estimation method^7^ consisting of five steps (see below and further details in the Appendix). Briefly, the method fits and validates a predictive statistical model trained on ground survey data and incorporating various predictor variables. The model is used to estimate both actual and counterfactual (no drought crisis) death rates, and multiplied by district-level population denominators to generate absolute and excess death tolls. For this analysis, we refined the method through a more comprehensive selection of statistical models and dynamic reconstruction of the population.

### Step 1: ground mortality data

In Somalia, most ground mortality data come from SMART (Standardised Monitoring of Relief and Transitions) surveys.^8^ These small-scale surveys, chiefly designed to assess acute malnutrition prevalence and nearly always relying on multi-stage cluster sampling, are conducted by various humanitarian actors. They often include a mortality module that captures household-level demographic changes over a relatively short (approximately 3–5 months) recall period.

Before 2016, most SMART surveys used an aggregate mortality questionnaire format, which was subsequently replaced with an individual-level format. Both formats enable estimation of the crude (all-ages, all-cause) and under 5 years death rate (CDR, U5DR), which we express as deaths per 10,000 person-days, the unit most familiar to humanitarian actors. While for many SMART surveys the sampling universe is a single district, coinciding with our analysis stratification, some surveys are conducted at the livelihood zone level (i.e., a region potentially encompassing multiple districts and characterised by a dominant economic activity, e.g., pastoralism). While we had previously excluded these surveys, here we include all livelihood survey clusters whose location (district) could be resolved using geographic information systems. This geolocation process involved establishing the spatial correspondence between reported place names and an official national register,^9^ supplemented by expert validation and manual completion of missing entries by a team from SIMAD University, Mogadishu.

Each included survey dataset underwent systematic curation. We excluded implausible values (e.g., negative death counts), recalculated key mortality indicators (CDR and U5DR), and assessed survey quality using a composite score (range: 0–1). While mortality data offer little insight into systematic or unsystematic error (unusual patterns could plausibly reflect local drivers of mortality or other demographic factors), we adopted a score widely used by the SMART initiative and composed from several indicators, such as normality and digit preference, calculated from the anthropometric component of the survey, which we reasoned would be a proxy of overall survey quality. Details on the score are provided in the Appendix (Figure S3). We used the score as an importance weight for survey observations entered into statistical models. Further details on how surveys are curated, inclusion the computation of person-time at risk, are provided elsewhere.^7^

### Step 2: population reconstruction

The last census in Somalia took place in 1975. Since then, large-scale internal displacement and out-migration have occurred. Therefore, we reconstructed population estimates by district and month to provide accurate denominators for death toll estimation and to scale predictor values to the population. Details of our reconstruction method are published separately.^10^ Briefly, we drew on our four alternative population sources disaggregated by district, including Afripop/WorldPop remote sensing estimates (January 2015),^11^ Expanded Programme on Immunisation (EPI, January 2019) planning data, household counts done by the Polio Eradication Initiative (December 2018) and the United Nations Population Estimation Survey of Somalia (UNPESS, March 2014).^12^ There are significant variations in these estimates.^10^ Notably, the Polio 2018 and EPI 2019 sources omit Baydhaba district and Bu'aale, Jilib and Saakow districts, respectively. The UNPESS also provided baseline (January 2015) IDP proportions per district, which we assumed to remain stable during 2015, a relatively drought-free year. From January 2016 to December 2018, we sourced publicly available data on incident internal displacement from the Protection and Returns Monitoring Network (PRMN),^13^ managed by the United Nations High Commissioner for Refugees (UNHCR). In addition, we reviewed UNHCR situation reports on the ReliefWeb information platform (www.reliefweb.int) and the UNHCR website (www.unhcr.org) to approximate refugee movements by district, including returns. Beyond December 2018, we assumed negligible refugee movements apart from Laas Canood district, where an estimated 80,000 people crossed into Ethiopia in February 2023.^14^ Using these data, and in contrast to the previous study,^6^ we developed a dynamic, mechanistic model of population growth and movement, including IDP returns to districts of origin. We used publicly available cross-sectional assessments conducted by the International Organisation for Migration's Displacement Tracking Matrix (DTM), albeit with incomplete geographic coverage, as ground observations to fit the model to and estimate unknown parameters governing IDP flows (proportion of IDPs who eventually return; rate of return) by region of Somalia.

### Step 3: predictors of mortality

We systematically searched both public and non-public sources to identify predictors, ideally specified at the district–month scale, that align with a theoretical causal framework of excess mortality in crisis settings.^15^ We retained predictors with a satisfactory level of completeness (≥70% of district-months) (see Appendix, Step 3).

### Step 4: predictive model

We combined predictor and ground mortality data into statistical models to predict all-age and under-5 years deaths per household during the recall period of each SMART survey, using the natural logarithm of household person-days at risk as an offset. We sought models (i.e. combinations of different predictor variables) that would perform well out of sample.

We explored various modelling approaches, including generalised linear models (GLMs), generalised additive models (GAMs) incorporating smoothing functions of each continuous predictor,^16^ and random forest (RF)^17^ algorithms. We fit both fixed-effects and mixed GLM models, the latter (GLMM) with district as a random effect to account for the longitudinal nature of the data. To identify the most preformant model, we quantified the root mean square error,^18^ bias and variability of model predictions relative to observed deaths using leave-one-out cross-validation (LOOCV).

Two separate models were developed: one for CDR and one for U5DR. These outcomes were modelled independently rather than jointly but using the same set of candidate predictors and lag structures for both models. Based on RMSE and bias measures, the most promising predictive model was a GLMM with survey observations weighted by the quality score. The model's formula is thus:

$$\log\;d_{i}=\sum_{j\;=\;1}^{j\;=\;P}x_{j,k,\delta }\beta_{j,\delta }\;+\;u_{k}\;+\;\log\;{\;\Pi }_{i}\;+\;\epsilon_{i}$$

where $d_{i}$ is the number of (under 5 years) deaths in household i during the survey's recall period; $x_{j,k,\delta }$ is the mean value of predictor j (for j∈P, where P is the set of model predictors) within the same district k as that of household i during the survey's recall period, potentially lagged by δ months; $\beta_{j,\delta }$ is the corresponding fixed-effect linear coefficient; $u_{k}$ is a random effects for district k, assumed to follow a normal distribution with mean 0 ($u_{k}\;∼\;N(0,{\sigma_{u_{k}}}^{2}$)), and capturing the repeated nature of observations; $\log\;\Pi_{i}$ is an offset to account for varying household (under 5 years) person-time Π at risk; and $\epsilon_{i}$ is the residual error not explained by the model. The survey's cluster design is not explicitly modelled, but a quasi-Poisson distribution of death counts per household is assumed to account for plausible overdispersion due to intra-cluster correlation, and robust standard errors are calculated. See Appendix, Step 4 for additional methods and comparisons of model performance: while overall bias was low, the model generally under-estimated observations during 2018 and exhibited inaccuracy in Woqooyi Galbeed and Bay regions.

The final models for both CDR and U5DR feature the following predictors of mortality: composite drought index, incidence of admissions for malnutrition to therapeutic feeding programmes, and incidence of suspected measles and malaria in outpatient health facilities.

### Step 5: estimate excess death tolls

We applied the models to predict CDR and U5DR by district-month, which we multiplied by population size to estimate death tolls, aggregated spatiotemporally as desired. Based on countrywide trends, we identified two distinct periods (January 2015–July 2016 and January 2020–July 2021) featuring relatively low death rates and coinciding with non-drought conditions. We adopted the district-specific death rates during these periods as alternative counterfactuals, namely mortality levels that would realistically have occurred in the absence of drought conditions. We subtracted these counterfactuals from model-estimated mortality to yield estimates of excess mortality. In previous studies,^7^ we defined the counterfactual by assuming specific values for the predictors in the absence of the crisis. In this analysis, which sought to estimate *drought-attributable* excess deaths specifically, plausible counterfactual predictor levels would have necessarily been set by adopting averages during non-drought periods: this, however, amounts to the same as non-drought death rates. The alternative counterfactual basis periods (2015–2016, 2020–2021) represent the extremes of non-drought mortality given model estimates over the analysis period, and thus provide a plausible counterfactual range.

To propagate uncertainty, we implemented 1000 simulations of the above computation, sampling with replacement from the uncertainty distributions of the district-month death rate estimates and population denominators. We present below the median and 95% percentile intervals of these runs.

All analyses in this paper were done in R using packages glmmTMB for mixed linear models, rfPoisson for random forest and gamlss or mgcv for additive modelling.

### Ethics

The Ethics Committee of the LSHTM (ref. 15334) and the Ethics Committee of the Ministry of Health and Human Services of Somalia (ref. MOH&HS/DGO/1485/November/2022) approved the study. Informed consent was not applicable because the study used secondary, anonymised data.

### Role of the funding source

The funders of the study had no role in study design, data collection, data analysis, data interpretation, or writing of the report.

## Results

### Availability of ground mortality survey data

In a previous analysis,^6^ 201 SMART surveys were available for the period 2013–2018, but only 97 were useable, mainly because many surveys were designed to be representative of livelihood zones rather than districts, with unresolved locations of survey clusters. In this study, 530 SMART surveys were available for the periods 2013–2024, and 496 were included in the analysis, reducing the exclusion rate from 52% to 6%. Despite this, survey clusters appeared to systematically omit densely populated parts of southern and central Somalia, plausibly due to access constraints (Fig. 1a). Survey coverage across time for each administrative region is shown in Fig. 1b. The median survey quality score was 92% (interquartile range, IQR 88%–96%), with a median recall period of 93 days (IQR 90–97) and a median of 5·3 (IQR 4·9–5·7) household members included per survey. Trends in CDR and U5DR as estimated in surveys are shown in the Appendix, Step 1.

**Fig. 1:**
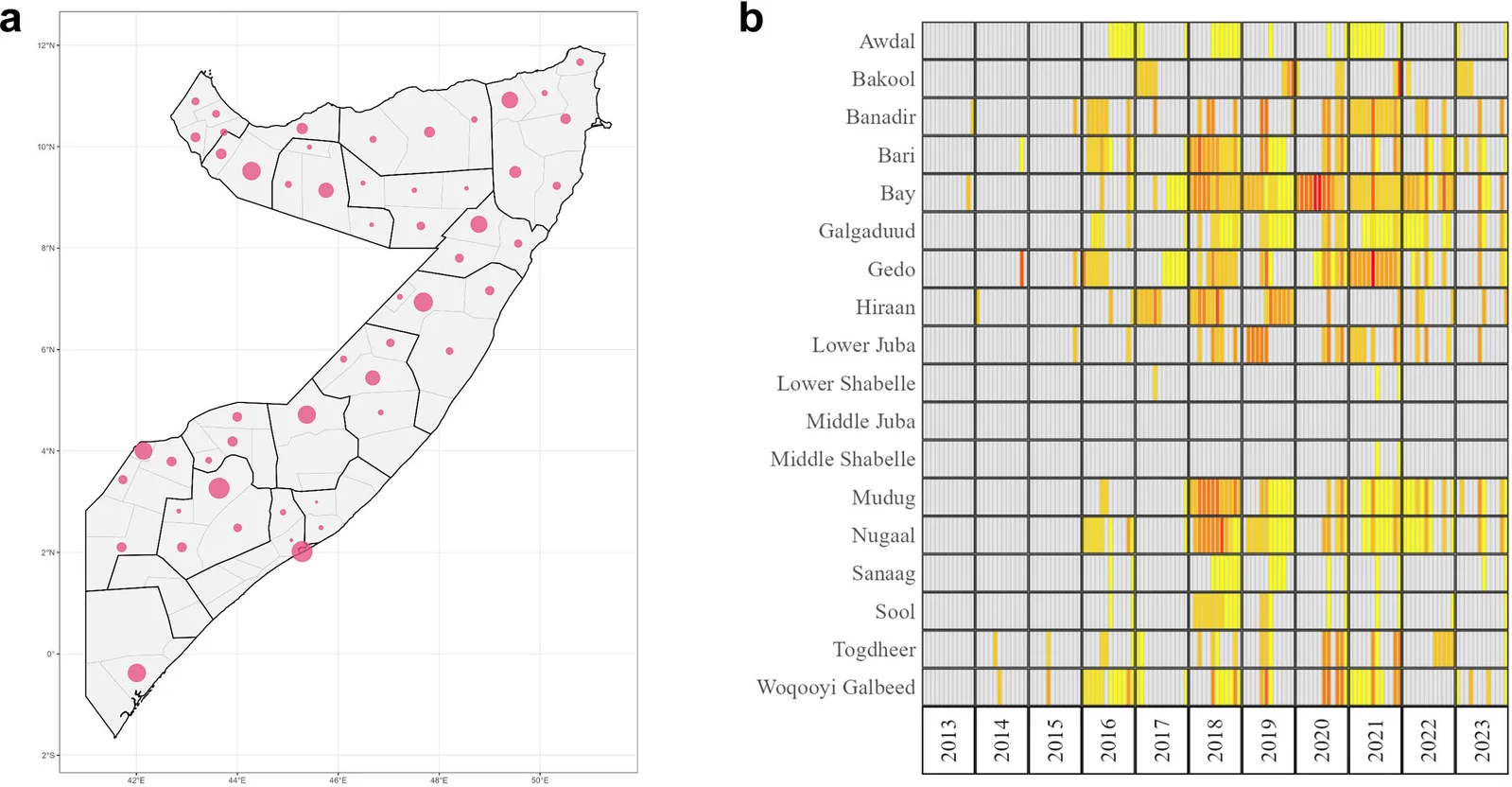
a) Map of SMART survey cluster locations. Each pink dot represents a location where SMART surveys were conducted, and the size of the dot represents the cluster size. b) Heat map showing the recall period coverage of SMART mortality surveys by region and month within the period of analysis. Colours in the yellow-red scale indicate the relative amount of information. Grey cells indicate regions and months that none of the surveys' recall period fell within.

### Predictor patterns

Drought conditions appear to have intensified over the 2017–2019 and 2022–2024 periods, coinciding with troughs in terms of trade (especially involving cereal-to-livestock exchange), an increase in the malnutrition admissions incidence, and peaks in cholera and measles cases (Appendix, Figure S5). In contrast, the incidence of insecurity-related events appears to have remained broadly stable throughout the study period.

### Excess mortality estimates

Fig. 2 illustrates the evolution of the estimated CDR at the countrywide level over the analysis period. We estimate 612,600 deaths (95% CI: 428,100–909,300) among all ages combined between January 2022 and June 2024, out of an estimated mid-period population of about 18·9 million.

**Fig. 2:**
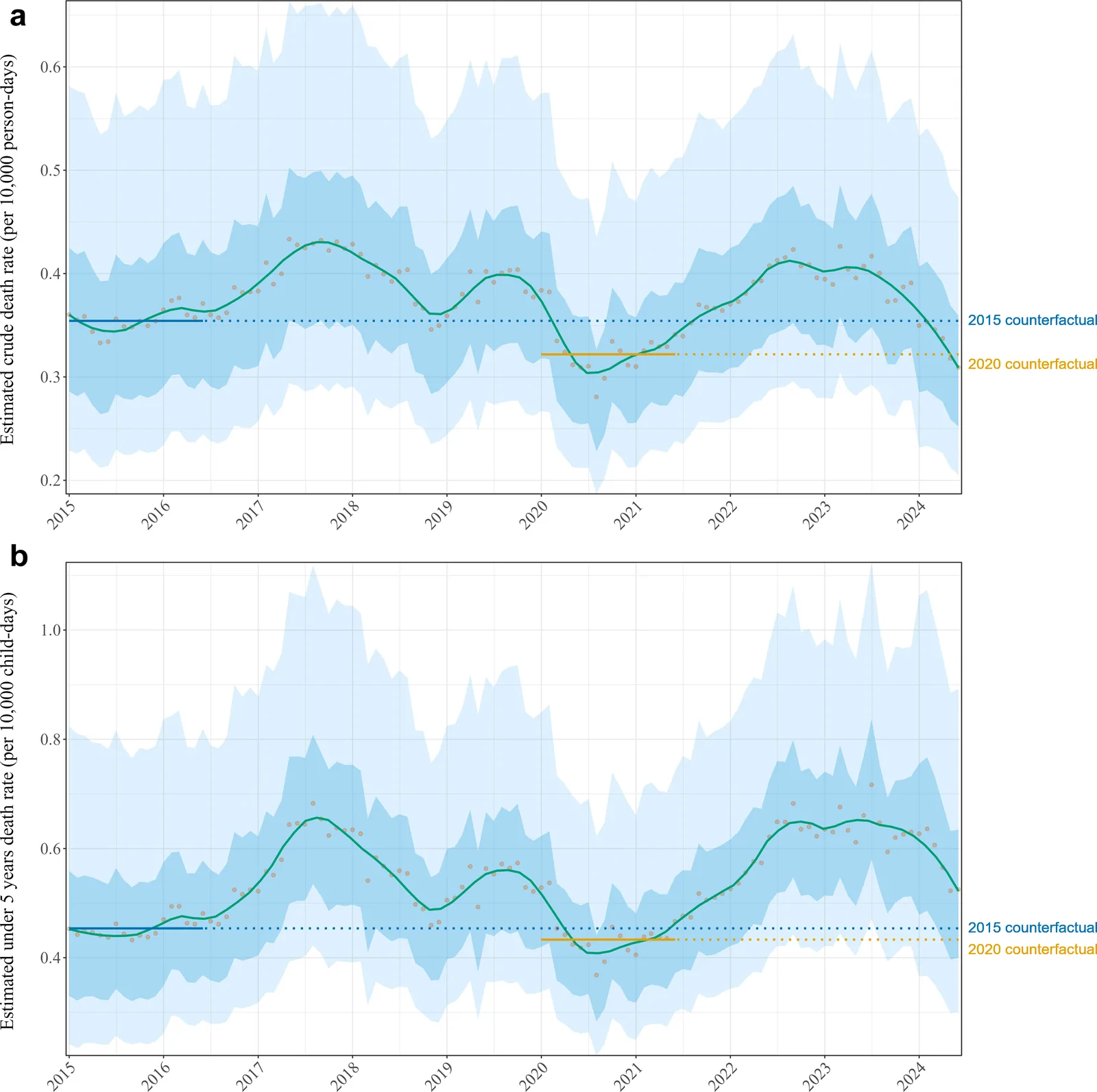
a) Estimates of the crude death rate per 10,000 person-days for Somalia as a whole, by month. b) Estimates of the under 5 years death rate per 10,000 person-days for Somalia as a whole, by month. Orange dots show the monthly median estimates and blue-shaded areas the 80% and 95% confidence intervals, with a locally weighted regression trend line shown in green. Lastly, the two alternative counterfactual baselines are depicted in blue and yellow, respectively.

Under the 2015–2016 counterfactual baseline, we estimate 71,100 excess deaths (95% CI 28,200–125,100) from January 2022 to June 2024, with 64,500 (95% CI 19,400–95,900) occurring among children under 5 years old. Alternatively, assuming a counterfactual based on 2020 trends, projected excess deaths increase to 104,700 (95% CI 17,000–170,300), with 73,200 (95% CI 29,200–105,700) among children under 5 years old.

Most excess mortality was projected to have occurred in Bay, Banadir, and other southern regions, including Lower Shabelle and Gedo. Geographically, the highest mean CDRs during 2022–2024 were estimated for districts in south-central Somalia, with Bay, Gedo, and Banadir exhibiting the highest values (Fig. 3, Table 1).

**Fig. 3:**
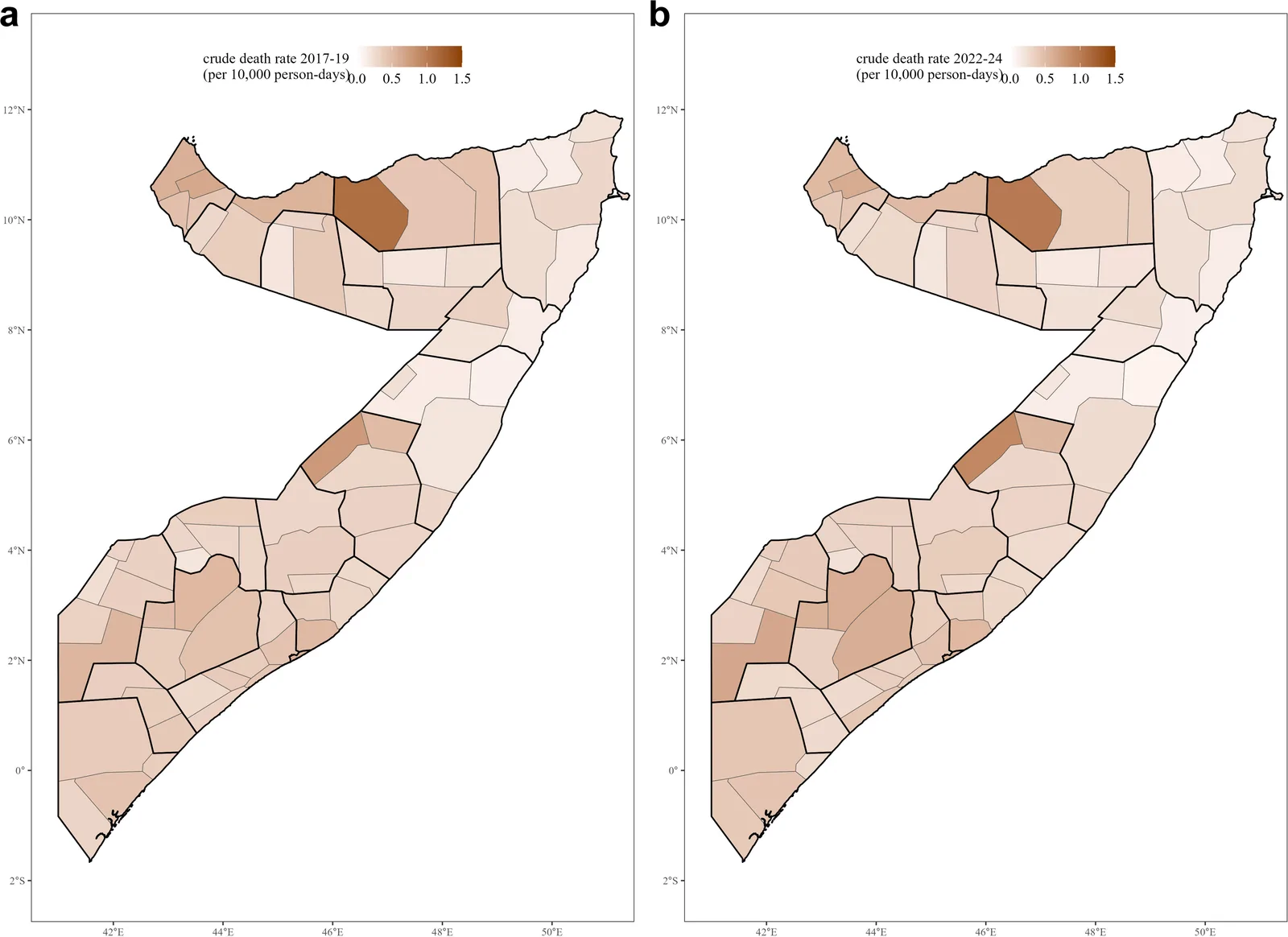
Mean estimated crude death rate per district during two previous drought periods (a: 2017–2019 and b: 2022–2024). Thick lines denote borders of regions (administrative level 1).

**Table 1:** Estimated excess deaths among all ages and children under 5 y old, by region, based on the 2015–2016 counterfactual level (rounded to the nearest hundred) using a GLMM model. Death tolls are presented for 2022, 2023 and the first half of 2024.

Region	January 2022–December 2022		January 2023–December 2023		January 2024–June 2024	
	All ages	Children under 5 y old	All ages	Children under 5 y old	All ages	Children under 5 y old
Awdal	−2300 (−4100 to −700)	−400 (−1000 to 0)	−2900 (−4700 to −1300)	−500 (−1100 to −100)	−1700 (−2600 to −1000)	−100 (−400 to 100)
Bakool	1200 (0–2000)	600 (100–1100)	1500 (100–2400)	800 (100–1300)	200 (−400 to 600)	200 (−100 to 400)
Banadir	14,400 (12,200–16,500)	9700 (8500–11,600)	14,600 (12,300–16,900)	11,200 (10,000–13,400)	−600 (−2500 to 1100)	3700 (1900–5600)
Bari	200 (−700 to 900)	300 (−300 to 700)	0 (−900 to 700)	100 (−400 to 600)	−200 (−600 to 200)	300 (−100 to 500)
Bay	6800 (5500–8000)	5500 (4700 to 6500)	8700 (6800 to 10,500)	8200 (6900 to 9700)	1500 (500–2400)	2800 (2000–3800)
Galgaduud	2900 (1100–4300)	1300 (500–2200)	3500 (1500–5000)	1600 (600–2400)	200 (−700 to 900)	400 (−200 to 700)
Gedo	3100 (1000 to 4600)	1800 (900–2800)	4400 (1900–6200)	2800 (1600–4100)	−300 (−1500 to 700)	500 (−200 to 1100)
Hiraan	1900 (0–3200)	800 (0–1400)	2200 (−100 to 3700)	1200 (200–1900)	−400 (−1700 to 500)	100 (−600 to 700)
Lower Juba	1500 (−1000 to 3100)	900 (−100 to 1500)	2100 (−600 to 3800)	1500 (400–2300)	0 (−1100 to 800)	600 (100–1200)
Lower Shabelle	4000 (−3300 to 8700)	1800 (−700 to 3700)	3700 (−3300 to 8300)	1800 (−700 to 3600)	400 (−2800 to 2500)	800 (−500 to 1700)
Middle Juba	400 (−1500 to 1500)	100 (−500 to 500)	200 (−1700 to 1400)	100 (−600 to 500)	0 (−1100 to 600)	0 (−400 to 200)
Middle Shabelle	1700 (−1900 to 4200)	1000 (−600 to 2200)	1500 (−1900 to 3900)	1400 (−400 to 2500)	0 (−1600 to 1000)	600 (−300 to 1100)
Mudug	700 (−500 to 1600)	500 (0–1000)	500 (−600 to 1300)	400 (−100 to 800)	−100 (−700 to 300)	100 (−200 to 300)
Nugaal	−400 (−800 to −100)	−100 (−400 to 100)	−900 (−1300 to −500)	−400 (−700 to −100)	−300 (−500 to −100)	0 (−200 to 200)
Sanaag	600 (−800 to 1700)	300 (−300 to 700)	500 (−800 to 1700)	300 (−300 to 700)	−200 (−1000 to 400)	200 (−200 to 400)
Sool	200 (−700 to 800)	100 (−200 to 400)	−100 (−800 to 500)	100 (−300 to 300)	−200 (−600 to 100)	0 (−200 to 100)
Togdheer	−800 (−1900 to 0)	−200 (−700 to 100)	−300 (−1500 to 700)	−100 (−600 to 300)	−500 (−1100 to 0)	−100 (−300 to 200)
Woqooyi Galbeed	100 (−1100 to 1300)	0 (−400 to 400)	−800 (−2000 to 300)	−100 (−500 to 300)	−1400 (−2100 to −800)	0 (−300 to 200)
Total	36,200 (1700–61,600)	24,000 (9600–36,900)	38,500 (2500–65,600)	30,400 (14,200–44,300)	−3600 (−22,000 to 10,300)	10,100 (−100 to 18,400)

Negative excess mortality signifies that death rates estimated under observed conditions were lower than the counterfactual level.

### Comparison with the previous drought crisis

Results for 2022–2024 are consistent with those observed during the 2017–2018 drought, when an estimated 519,100 deaths (95% CI 352,800–794,300) occurred among all age groups. Under the same 2015–2016 counterfactual baseline, 79,600 excess deaths (95% CI 8200–129,600) were estimated between January 2017 and June 2019, of which 49,000 (95% CI 6500–69,800) or 61% among children under 5 y old (see Appendix, Step 5 for estimates by region).

Fig. 2 suggests that the period of elevated CDR temporally associated with the ongoing drought crisis began in January 2022 and ended in June 2024, corresponding to 30 months of excess mortality. To benchmark the relative severity of the past two droughts, Table 2 compares mortality metrics during the first 30 months of each. This crude comparison suggests that the most recent drought crisis was similar to the previous one in mortality terms. However, compared to the population 5 years earlier, Somalia was projected to have increased by nearly 3 million people, which, even for the same excess death rate, would result in a higher death toll.

**Table 2:** Comparison of different estimated mortality indicators during the 30 months of the latest two drought-triggered crises in Somalia.

Indicator	January 2017–June 2019	January 2022–June 2024
CDR (all ages)	0.37 (95% CI: 0.24–0.61)	0.36 (95% CI: 0.22–0.56)
Highest estimated CDR (per district)	0.60 per 10,000 person-days	0.99 per 10,000 person-days
Difference between minimum and maximum CDR	0.41 per 10,000 person-days	0.41 per 10,000 person-days
Regions with the highest three CDR levels (CDR point estimate)	Banadir (0.59), Sanaag (0.55), Bay (0.49)	Banadir (0.57), Bay (0.55), Sanaag (0.48)

## Discussion

This ten-year retrospective study finds that two drought-triggered crises, occurring only five years apart against a background of chronic insecurity, resulted in large-scale excess mortality across Somalia, with deaths concentrated among young children and in the country's south-central regions. Notably, we estimate that the two drought crises caused a similar number of excess deaths. Although the death tolls and peak death rate did not approach the levels seen during the 2010–2012 famine, they indicate persistent unmet needs in both climate resilience and humanitarian response. Beyond the Somali context, our analysis helps to refine statistical methods for estimating mortality using a small area estimation approach in settings where civil registration is not a robust source, but where various alternative and mostly previously collected data sources can usefully be combined.

Mortality in other recent droughts is not well-established, often reflecting limited sharing and accessibility of data^19^ and the difficulty of attributing the cause of death to drought in poly-crises where armed conflict is a key driver. By way of comparison, Somalia's 2010–2012 famine caused about 256,000 deaths, around half among children under 5 y.^2^ In a context of war-triggered food insecurity across South Sudan during 2014–2018, 192,000 non-violent excess deaths were estimated, with CDR peaking around 0.8 per 10,000 person-days,^20^ while in northeast Nigeria 490,000 excess deaths and a peak CDR of 0.8 per 10,000 person-days were estimated during 2016–2019, of which an unknown proportion violent injuries.^21^ Additional evidence of high excess mortality, especially for under 5-years, and of its proximate biomedical causes is provided by prospective surveillance of selected IDP sites in Somalia. These analyses consistently documented extremely high prevalence of acute malnutrition, measles outbreaks, and diarrhoeal diseases, particularly among children under 5 years, during both drought periods,^22^^,^^23^ exacerbated by poor water, sanitation and hygiene (WASH) infrastructure, low vaccination coverage, and severely constrained access to curative health services (31, 33). In IDP settlements within Mogadishu, CDR and U5DR were 2.3 and 5.2 per 10,000 person-days in the immediate post-displacement phase of 2022–2023, but declined thereafter, with comparable estimates in IDP settlements outside the capital region^24^; verbal autopsies suggested that severe malnutrition was the top cause of deaths.^23^ Taken together, these alternative estimates reinforce our modelling results.

It may however be surprising that no excess mortality was evident during the COVID-19 pandemic period in this analysis, although we previously estimated using satellite imagery of cemeteries an approximately 50% increase in burials from baseline in Mogadishu during the first SARS-CoV-2 wave, with some 4000–11,000 excess deaths.^25^ As shown in Figure S11 (Appendix), no obvious increase in CDR levels estimated by surveys is seen during the pandemic period, including in urban settings. We also speculate that the set of predictors we considered here would not have enabled the detection of a pandemic peak, as they are unlikely to bear a causal relationship with COVID-19. There remains considerable uncertainty about mortality from COVID-19 in different Sub-Saharan African settings,^26^ though serious elevations have been measured in specific settings.^27^ It is possible that, as seen elsewhere in Sub-Saharan Africa,^28^ COVID-19 mortality in rural areas of Somalia was relatively mild, reflecting a young population and environmental factors favouring low transmissibility.

By contrast, excess mortality appeared closely coupled, albeit with a few months’ delay, with measures of a composite index of crisis-emergent risk factors,^29^ as illustrated in the Appendix, Figure S9. This delay is biologically plausible, has been observed in previous droughts^1^ and likely reflects the unfolding of causal chains from food insecurity to depletion of coping mechanisms, insufficient nutrient intake, onset and progression of malnutrition, occurrence of individual- and population-level complications (e.g. epidemics of severe infectious disease) and, finally, mortality. This temporal concordance suggests that there is value in reinforcing anticipatory action based on early warning indicators of an impending crisis.

It may also be important to note the relatively lower excess mortality estimated in north-eastern regions despite high levels of food insecurity. Although the statistical models do not explicitly explain this pattern, several factors may have moderated mortality risk, including predominantly pastoral livelihood systems with greater mobility and coping capacity, differences in the timing or coverage of humanitarian assistance, and lower population density and baseline disease burden. In addition, mortality estimates for some northern regions are based on fewer surveys, resulting in greater uncertainty and reduced power to detect moderate mortality increases. Generally, the region-specific estimates suggest considerable heterogeneity in drought impacts. The large number of excess deaths in Banadir reflects this region's disproportionately large population and high concentration of internally displaced persons.^30^

At the same time, these findings also point to the limits of what early warning systems and anticipatory humanitarian action can achieve in isolation. The persistence of drought-related excess mortality at levels comparable to those observed during the 2017–2018 crisis, despite improvements in data availability, coordination, and response timeliness, indicates that technical and operational gains alone have not been sufficient to reduce mortality risk. Conceptual analyses of humanitarian action have highlighted how assistance can play an important stabilising role in contexts of chronic crisis, while remaining structurally constrained by the political, economic, and institutional environments in which it operates.^31^ Similarly, evidence from climate adaptation research suggests that externally driven interventions may have variable effects on vulnerability when they are not closely aligned with local institutional capacity, power relations, and livelihood systems.^32^ Qualitative studies from other disaster-affected contexts further indicate that humanitarian practices may interact with existing social and institutional dynamics in ways that shape their effectiveness over time.^33^

### Limitations

Our estimation process relies on multiple components, each introducing potential error. Confidence intervals propagate three sources of unsystematic error. (i) variability in survey-observed death rates at household level, which could only be improved upon through more frequent, larger surveys; (ii) random noise and missingness in the predictor district-month time series (notably, there were gaps in malaria data from Mudug, Bakool, and Sanaag), and/or an insufficient set of predictors (access to a greater range of predictors within the theoretical causal framework of mortality could have enhanced model performance; in particular, robust data on public health and humanitarian service provision, e.g. outpatient and inpatient service utilisation, vaccination coverage and receipt of food and/or cash transfer interventions to improve food security, were not available despite substantial efforts to identify or update such datasets; this highlights the need for more systematic data collection and sharing within the humanitarian response); (iii) empirical uncertainty distributions of district-month population estimates, which could be mitigated through census-like activities and improved displacement and return tracking.

The results may also be affected by systematic error. Firstly, bias in the SMART survey ground mortality observations could affect our analysis. While previous analyses have not shown patterns suggestive of death rate underestimation as a function of type of community surveyed,^6^ our addition of livelihood zone-based surveys in this study may introduce bias. Survey estimates conducted at these different administrative levels show consistent differences, with U5DR lower in livelihood zone surveys (Appendix, Figure S2) possibly due to challenges with accessing the full sampling universe or interviewer familiarity with local contexts. Data quality issues have been observed in Demographic and Health Surveys clusters as a function of distance from urban areas, further reinforcing the potential for underestimation.^34^

Secondly, systematic errors in the predictors might not affect estimates if consistent across time and space, but non-systematic errors (equivalent to signal noise) could lead to underestimation of corresponding model coefficients, known as ‘regression dilution’.^35^

Thirdly, despite improved methods, population estimates remain uncertain due to disparate data sources and methods for tracking IDPs and refugees, often relying on key informant estimation. It is, however, difficult to speculate about the directionality of any potential bias. Overestimation of out-migration from heavily drought-affected regions where mortality appeared particularly high (e.g. Bay, Bakool and Lower Shabelle) would have resulted in under-estimation of the denominator remaining at risk within those regions, and thus excess mortality—and vice versa. Data on race/ethnicity and gender were not available for reporting.

Fourthly, limited or altogether missing geographic coverage of survey clusters may have reduced the model's performance in under-surveyed settings and contributes to uncertainty in fine-scale spatial estimates. A small-area estimation predictive model should be able to cope with geographic gaps in training data coverage without incurring bias, as long as (a) the relationship between predictors and the outcome is the same across geography and (b) the full range of possible values of the predictors and the outcome is captured by available training data. While neither condition can be verified directly, maps of drought/food insecurity produced independently from this analysis^36^ suggest that the areas most affected (mainly south-central Somalia) were adequately covered by survey clusters; the surveys at our disposal also offer diversity in terms of periods and livelihood zone, and include urban as well as IDP populations. As such, we suspect that survey coverage gaps would have mainly contributed to random noise/non-systematic error.

Finally, CDR and U5DR were modelled separately, using the same set of predictors and lag structures, rather than within a joint age-structured mortality framework. This approach was chosen to maximise predictive performance given available data, but it does not explicitly model age-specific causal pathways linking drought, disease exposure, nutritional status, and mortality risk. As a result, apparent age differentials, particularly the concentration of excess mortality among children under five, may be accentuated if predictors affect age groups differently or if unmeasured protective factors operate unevenly across ages. Considering these limitations, we urge readers to interpret the findings within the context of Somalia and in light of other available evidence on the drought crisis’ effects. Overall, we cautiously assess that the balance of the above error sources is likely to result in underestimation rather than overestimation. Sensitivity analyses in the Appendix explore potential drivers of bias.

### Conclusions

In Somalia, the combination of intensifying droughts attributable to climate change in the Horn of Africa, substantially declined global humanitarian assistance budgets, and steep population growth threaten to cause persistent and potentially worsened health impacts over the coming decades, including a large number of lives lost. Against this prospect, building climate resilience at community and institutional levels seems essential, through investments in sustainable water management, drought-resistant agriculture, and adaptive social protection systems that can buffer vulnerable populations against recurrent shocks.^37^, ^38^, ^39^ What humanitarian resources are left will need to be targeted efficiently; as shown here, crisis impacts feature considerable district-level disparities, plausibly with further inequality at an even more granular scale. To support spatiotemporally accurate analysis, standing mechanisms to monitor food security and public health threats, including mortality and key upstream causal factors, such as IPC should be enhanced rather than defunded. Other adaptations have been suggested: greater use of unconditional cash transfers as an all-purpose intervention to support food-insecure households; localisation of assistance by civil society, as advocated for Somalia^40^ and other crises^41^; and leveraging the already-critical contribution of diaspora remittances to fill some of the gaps left behind by withdrawn humanitarian aid.^42^^,^^43^ Perhaps most importantly, national reconciliation and peacebuilding would remove a key complicating factor standing in the way of Somali-led progress.

## Contributors

F.C. conceived and designed the study. Y.O., D.A., Y.A., M.A., N.D., A.W., M.Y., C.G., O.W., and F.C. contributed to data acquisition and had access to the raw data. Y.O., D.A., M.Y., C.G., O.W., and F.C. verified the underlying data. Y.O., O.W, D.A., M.Y. and F.C. conducted the formal analysis and interpreted the results, with input from all authors. Y.O. draughted the first version of the manuscript. All authors critically reviewed, revised, and approved the final version of the manuscript. Y.O. and F.C. had final responsibility for the decision to submit for publication.

## Data sharing statement

Data and analysis code are available at https://github.com/afyac/mortality_estimation.

## AI use statement

Artificial intelligence was used to assist with English language editing and refinement of the manuscript. Specifically, the authors used ChatGPT to improve clarity, grammar, and readability. Prompts provided to the tool included requests to revise text for academic style, improve sentence structure, and align sections with journal guidelines (e.g., “revise for Lancet style”, “improve clarity and conciseness”, and “edit for grammar and flow”). All outputs were carefully reviewed and validated by the authors, who take full responsibility for the content of the manuscript.

## Declaration of interests

C.G. reports grant funding from the World Health Organization and UNICEF related to this work. These grants funded the research and supported 0.1 full-time equivalent salary for 1 year, with payments made to the London School of Hygiene & Tropical Medicine, which paid C.G.’s salary as an employee. All other authors declare no competing interests.

## Appendix A Supplementary data

Appendix

EPD form
