# Supplementary material for: Drought-attributable mortality in Somalia, 2015–2024: a retrospective and modelling study
Source: Lancet Reg Health Afr. 2026 Oct;8:None. doi: 10.1016/j.lanafr.2026.100072 (PMC13672283; doi:10.1016/j.lanafr.2026.100072)
Supplement: Supplementary file 1 [file mmc2.pdf]

Equitable Partnership Declaration

If any questions do not apply to your study, please indicate "N/A" for "not applicable.
For more information on how to complete this form see the Information for Authors document.

Researcher considerations

1. Please detail the involvement that researchers who are based in the country or countries of study had during a) study design; b) clinical study processes, such as processing blood samples, prescribing medication, or patient recruitment; c) data interpretation; and d) manuscript preparation, commenting on all aspects. If they were not involved in any of these aspects, please explain why.

   This should include a thorough description of their leadership roles in the study. Are local researchers named in the author list or the acknowledgements, or are they not mentioned at all (and, if not, why)? Please also describe the involvement of early career researchers based in the location of the study. Some of this information might be repeated from the Contributors section in the manuscript. Note: we adhere to ICMJE authorship criteria for naming authors on a paper.

**a) Study design:**<br>Retrospective observational and statistical modelling study using secondary data sources.
**b) Clinical study processes:**<br>Not applicable. This study did not involve primary data collection, clinical procedures, patient recruitment, biological sample collection, or interaction with participants. All analyses were based on secondary data from existing surveys and administrative datasets.
**c) Data interpretation:**<br>Researchers based in Somalia (D.A., Y.A., M.A) and internationally (Y.O, A.W., C.G., O.W, F.C) contributed jointly to data interpretation. Somalia-based co-authors provided contextual expertise on demographic patterns, humanitarian conditions, displacement dynamics, and health system constraints, which informed interpretation of spatial and temporal mortality patterns.
**d) Manuscript preparation:**<br>Manuscript drafting was led by the first and senior authors, with iterative input from all co-authors. The first author is an early career researcher. Researchers based in Somalia (D.A., Y.A., M.A) contributed to interpretation of findings, contextual framing, and revision of manuscript drafts. All authors reviewed and approved the final version.

2. How was funding used to remunerate and enhance the skills of researchers in the countries of study? And how was funding used to improve research infrastructure at the study sites?

   Potentially effective investments into long-term skills and opportunities within local institutions could include training or mentorship in analytical techniques and manuscript writing, opportunities to lead all or specific aspects of the study, financial remuneration rather than requiring volunteers, and other professional development and educational opportunities.

Improvements to research infrastructure could include funding extended trial designs (eg, platform trials), establishment of long-term contracts for research staff, building research facilities, and setting up local control of funding allocation.

**Skills:**<br>Funding supported analytical capacity development through collaboration between international and Somalia-based researchers, including involvement in statistical modelling, data interpretation, and manuscript preparation. Somalia-based collaborators contributed to interpretation of findings and co-authorship of the manuscript, supporting skills development in quantitative epidemiology and population health analysis.
**Research infrastructure:**<br>No direct funding was allocated to physical infrastructure development at study sites. However, the study supported research capacity indirectly through strengthening analytical workflows, data integration systems, and collaborative research networks between Somalia-based institutions and international partners.

3. How did you safeguard the researchers who implemented the study?

Please describe how you guaranteed safe working conditions for study staff, including provision of appropriate personal protective equipment, protection from violence, and prevention of overworking.

The study involved secondary data analysis only and did not require fieldwork in insecure settings.

<u>Benefits to the communities and regions of study</u>

4. How does the study address the research and policy priorities of its location?

How were the local priorities determined and then used to inform the research question? Who decided which priorities to take forward? Which elements of the study address those priorities?

The study was informed by longstanding humanitarian and public health priorities in Somalia, particularly the need for improved mortality estimation in drought-affected settings.

5. How will research products be shared in the community of study?

For instance, will you be providing written or oral layperson summaries for non-academic information sharing? Will study data be made available to institutions in the region(s) of study? The Lancet Group *encourages authors to translate the summary (abstract) into relevant languages after paper editing; do you intend to translate your summary?*

The study data and code are publicly available via GitHub. Findings will be disseminated through peer-reviewed publication and may be shared with humanitarian and public health stakeholders in Somalia.

6. How were individuals, communities, and environments protected from harm?

a) *How did you ensure that sensitive patient data were handled safely and respectfully? Was there any potential for stigma or discrimination against participants arising from any of the procedures or outcomes of the study?*

All data used in this study were anonymised and aggregated at district or population level. No individual-level identifiers were available. There is minimal risk of stigma or discrimination arising from the study findings.

b) *Might any of the tests be experienced as invasive or culturally insensitive?*

Not applicable. No direct participant interaction or testing procedures were performed.

c) *How did you determine that work was sensitive to traditions, restrictions, and considerations of all cultural and religious groups in the study population?*

Study design and interpretation were informed by collaboration with Somalia-based researchers familiar with cultural, social, and religious contexts. No procedures involving cultural intrusion were undertaken.

d) *Were biowaste and radioactive waste disposed of in accordance with local laws?*

Not applicable.

e) *Were any structures built that would have impacted members of the community or the environment (such as handwashing facilities in a public space)? If so, how did you ensure that you had appropriate community buy-in?*

Not applicable.

f) *How might the study have impacted existing health-care resources (such as staff workloads, use of equipment that is typically employed elsewhere, or reallocation of public funds)?*

The study used only secondary data and did not directly affect health-care resources or service delivery.

7. Confirm that local ethics review was sought, and please provide the approval number. If not sought, please explain why.

Yes. Ethical approval was obtained from the Ethics Committee of the London School of Hygiene & Tropical Medicine (Ref: 15334) and the Ethics Committee of the Ministry of Health and Human Services of Somalia (Ref: MOH&HS/DGO/1485/November/2022).

Secondary analyses

8. Have the data analysed in your study been extracted from another source, such as a national survey, rather than being directly collected by the authors of this paper?

Yes / No / N/A

If the authors of this paper were not involved in data collection, how were the findings interpreted with sufficient contextual knowledge?
The Lancet Group *believe contextual understanding is crucial for informed data analysis and interpretation.*

The study used secondary data extracted from multiple sources, including population surveys and administrative datasets. Somalia-based co-authors contributed substantial contextual expertise regarding population structure, humanitarian conditions, and health system functioning, which informed interpretation of findings and ensured contextual validity of modelling outputs.

---

9. Please provide the title (eg, Dr/Prof, Mr/Mrs/Ms/Mx), name, and email address of an author who can be contacted about this statement.

Name: Yamna Ouchtar
Email: yamna.ouchtar@lshtm.ac.uk

10. Finally, please provide the title and name of an author from one country of study who has seen and approved this form.

Name: Najib Isse Dirie
