# Supplementary material for: Drought-attributable mortality in Somalia, 2015–2024: a retrospective and modelling study
Source: Lancet Reg Health Afr. 2026 Oct;8:None. doi: 10.1016/j.lanafr.2026.100072 (PMC13672283; doi:10.1016/j.lanafr.2026.100072)
Supplement: Supplementary file 2 [file mmc1.docx]

Drought-attributable excess mortality in Somalia, 2015-2024

**APPENDIX**

**Table of contents**

[Description of estimation steps	2](#_Toc224293345)

[Overall	2](#_Toc224293346)

[Step 1: Ground mortality data	2](#_Toc224293347)

[Step 3: Mortality predictors	4](#_Toc224293348)

[Step 4: Model evaluation	6](#_Toc224293349)

[Step 5: Estimate excess death toll	8](#_Toc224293350)

[Additional analyses	10](#_Toc224293351)

[Comparison between composite crisis index and mortality estimates	10](#_Toc224293352)

[Sensitivity analysis A: Impact of population denominator uncertainty	11](#_Toc224293353)

[Sensitivity analysis B: Impact of under-detection of under-5 deaths	12](#_Toc224293354)

[Trends in survey estimates over time	13](#_Toc224293355)

[References	15](#_Toc224293356)

## Description of estimation steps

### Overall

Figure S1 shows the estimation framework steps and required data inputs. We refined a small-area statistical estimation method previously implemented in 2022 (1) by modifying several steps:

- **Step 1:** Incorporated GIS methods to identify the district in which survey clusters were located and thereby include in the analysis mortality surveys targeting livelihood zones as their sampling universe (see below).
- **Step 2:** Estimated population denominators using a novel method that integrates multiple sources of census information, refugee in- and out-flows, internal displacement within and among districts and, critically, return of some displaced people to their communities of origin. Methods for this step are described fully in a separate paper (2) .
- **Step 4:** Evaluated a range of statistical and machine learning models, compared using Mean Squared Error (MSE) and Root Mean Square Error (RMSE).

Figure S1. Schematic of estimation framework and required data inputs.

### Step 1: Ground mortality data

We compiled raw and meta-data from surveys that estimated crude death rates (CDR) and under-five mortality rates (U5DR) conducted in Somalia between 2014 and 2023 among internally displaced persons (IDPs) or the general population. While most surveys targeted a given district (administrative level 2) as their sampling universe, some instead targeted a livelihood zone, i.e. a geographic area potentially crossing multiple district boundaries but characterised by a dominant livelihood (pastoral; agro-pastoral; agricultural; riverine) with a specific drought-related risk profile. Surveys typically had ‘recall’ (retrospective) periods of 3-4 months and relied on two-stage cluster sampling, with data on demographic changes during the recall period (death, birth, in- and out-migration) collected on each individual within households sampled, including those who died or left the household during the recall period. Details on how surveys are pre-processed, including the calculation of household person-time, are published separately(1). Figure S2 summarises annual patterns in survey estimates included in the analysis.

Figure S2. Box plots of crude and under 5 years death rates from surveys conducted in Somalia between 2014 and 2023, by survey sampling universe. Boxes show the median and inter-quartile range, whiskers the 95% percentile bounds and dots outliers.

For each survey, we computed a quality score based on data patterns observed in the anthropometry component of the survey and developed by the SMART Initiative. The score considers data characteristics including skewness, kurtosis and digit preference, and assigns penalties for apparent deviance in these metrics (Table S1). The different penalties are then summed, and quality score calculates using the following formula $quality score= 1-0.01\sum penalties$.

| Indicator | Statistic computed | Threshold | Penalty added |
| --- | --- | --- | --- |
| Flagged WHZ values | % of WHZ outside mean ±3 | >2.5-5% | +5 |
|  |  | >5-7.5% | +10 |
|  |  | >7.5% | +20 |
| Sex ratio test | Chi-squared test male vs. female | p-value: 0.05-0.1 | +2 |
|  |  | p-value: 0.001-0.05 | +4 |
|  |  | p-value: ≤ 0.001 | +10 |
| Age ratio test | Proportion test (6-29 vs. 30-59 months) | p-value: 0.05-0.1 | +2 |
|  |  | p-value: 0.001-0.05 | +4 |
|  |  | p-value: ≤ 0.001 | +10 |
| Digit preference  (for weight, height and MUAC) | Digit preference score | 8-12 | +2 |
|  |  | 13-20 | +4 |
|  |  | >20 | +10 |
| WHZ standard deviation | Standard deviation of WHZ (excluded flagged values) | 1.10-1.15 | +5 |
|  |  | 1.15-1.20 | +10 |
|  |  | >1.20 | +20 |
| WHZ skewness and kurtosis | Skewness and kurtosis statistics | ±0.2–0.4 | +1 |
|  |  | ±0.4–0.6 | +3 |
|  |  | >±0.6 | +5 |
| Cluster-level GAM distribution | Chi-squared test for Poisson distribution of GAM across clusters | p-value: 0.01-0.05 | +1 |
|  |  | p-value: 0.001-0.01 | +3 |
|  |  | p-value: <0.001 | +5 |

Table S1. SMART survey penalty score calculation.

Figure S3 shows the distribution of quality scores by survey sampling universe.

In previous analyses we have also included a ‘representativeness weight’ which accounts for issues such as survey clusters being skipped or replaced due to insecurity: however, we did not have sufficient detail to assess such bias given the meta-data available for Somalia.

Figure S3. Histograms of the quality score distribution among surveys conducted in Somalia between 2014 and 2023, by survey sampling universe.

### Step 3: Mortality predictors

We considered multiple predictors across climate, security, livelihood, malnutrition and health causal domains of mortality, selected because of their consistent availability by district and/or district-month. Pre-processing steps included dividing these by district population where appropriate, scaling and centring, applying temporal lags, smoothing times series through local splines to remove outliers (with a spar parameter = 0.4, corresponding roughly to moderate smoothing), and limited random forest-based imputation of missing values using the R mice package (3). Table S2 summarises predictors entered into candidate models. Trends in predictor values are shown in Figure S4.

Table S2. List and characteristics of predictors considered in model evaluation.

| **Predictor** | **Variable** | **Causal domain** | **Timespan of availability** | **Sources**  (Citations and URL if publicly available) | **Preprocessing steps** |
| --- | --- | --- | --- | --- | --- |
| Rainfall | mean of standard precipitation Index | Climate | 1982 to 2024 | Climate Engine  via API client for R package CHIRPS (4)  (<https://github.com/ropensci/chirps>) | Lags (4 to 6 months) |
| Extent of drought conditions | combined drought index | Climate | 1982 to 2024 | Somali Water and Land Information Management (SWALIM) project, Food and Agriculture Organisation  (<https://cdi.faoswalim.org/index/cdi>) | Lags (4 to 6 months) |
| Incidence of insecurity events and fatalities | events of any typology per 100,000 population  fatalities per 100,000 population | Exposure to armed conflict / insecurity | 2010 to 2024 | Armed Conflict Location & Event Data Project (ACLED) (5)  (<https://acleddata.com/data-export-tool/>) | Per-population rate |
| Incidence of attacks against aid workers | deaths per 100,000 population  injuries per 100,000 population | Exposure to armed conflict / insecurity | 2010 to 2024 | Aid Worker Security Database  (<https://aidworkersecurity.org/incidents>) | Per-population rate |
| Water price | price of 200L drum of water in Somali Shillings | Food insecurity and livelihoods | 2013 to 2024 | Food Security and Nutrition Analysis Unit for Somalia (FSNAU) (6) (<https://fsnau.org/ids/index.php>) | Smoothing, imputation, lags (2 to 4 months), adjusted for inflation based on January 2017 USD |
| Terms of trade purchasing power index | Kcal equivalent of local cereals that an average local-quality goat can be exchanged for | Food insecurity and livelihoods | 2013 to 2024 | FSNAU (6)  (<https://fsnau.org/ids/index.php>) | Smoothing, lags (2 to 4 months), imputation |
|  | Kcal equivalent of local cereals that can be purchased with an average daily labourer wage | Food insecurity and livelihoods | 2013 to 2024 | FSNAU (6)  (<https://fsnau.org/ids/index.php>) | Smoothing, lags (2 to 4 months), imputation |
| Malnutrition incidence | cases of severe acute malnutrition admitted to treatment programmes per 100,000 population | Nutritional status | 2013 to 2024 | Nutrition Cluster, Somalia | Per-population rate, lags (0 to 2 months) |
| Cholera incidence | cases per 100,000 population | Disease burden (epidemic) | 2015 to 2024 | FSNAU (6)  (<https://fsnau.org/ids/index.php>) | Per-population rate |
| Measles incidence | cases per 100,000 population | Disease burden (epidemic) | 2015 to 2024 | World Health Organization (unpublished) | Per-population rate |
| Malaria incidence | cases per 100,000 population | Disease burden (epidemic) | 2015 to 2024 | World Health Organization (unpublished) | Per-population rate |

Figure S4. Predictors completeness across time.

Figure S5. Trends in predictors with continuous values. The shaded areas represent the 2017-2018 and 2022-2023 drought periods. The ‘extent of drought conditions’ predictor (Combined Drought Index) takes low values during intense drought periods, and vice versa.

### Step 4: Model evaluation

For brevity, we do not show all model evaluation results. Table S3 below summarises the main models evaluated. Predictors included in the formulae for generalised linear (GLM) and generalised additive (GAM) models reflect brute-force searches across all combinations of candidate predictors to identify the highest-performing predictor set. GLM and GAM models modelled the outcome (deaths in the household during the survey’s recall period) assuming a quasi-Poisson distribution, with the log of person-time exposure in the household as an offset. P-spline smoothing terms were used for GAMs. Random forest (RF) models were grown using 1000 trees and a maximum of 3 variables to split data along at each tree node, with the ratio of household deaths to person-time as the outcome. Observations from each survey were weighted in models according to the quality score of each survey, scaled from 0 to 1 and computed as described above.

Model performance was evaluated using leave-one-out cross-validation (LOOCV), whereby one district-trimester’s worth of survey observations (fold) were left out of model training and model predictions for the fold were tracked, cycling through all possible folds and averaging model performance (mean square error, MSE, and root mean square error, RMSE) within each of the folds. This was done to identify models that performed well not just overall but also within different sub-periods of the overall timeframe of analysis.

Generally, models performed well in terms of bias, but there was evidence of overfitting (higher error on LOOCV than on the training data) for the RF model, as shown in Table S3 and Figure S6.

Table S3. Performance of alternative models evaluated.

| **Outcome** | **Model type** | **Features** | **Performance on training data** | | **Performance on leave-one-out cross-validation (LOOCV)** | |
| --- | --- | --- | --- | --- | --- | --- |
|  |  |  | RMSE† | Bias | RMSE† | Bias |
| Crude death rate | Generalised linear model (GLM) | Measles incidence (categorical), Combined Drought Index (lag 5mths), SAM admissions rate (lag 1mth) | 5.25e-06 | <1% | 5.00e-06 | <1% |
| Under 5 years death rate | Generalised linear model (GLM) | Terms of trade (wage to cereal) (lag 4mths), Combined Drought Index (lag 5mths), SAM admissions rate (lag 1mth) | 7.89e-06 | <1% | 7.65e-06 | <1% |
| Crude death rate and under 5 death rate | Generalised linear mixed model (GLMM) | Measles incidence (categorical), malaria incidence (categorical), Combined Drought Index (lag 5mths), SAM admissions rate (lag 1mth)  District as random effect | 5.56e-07 | <1% | 4.81e-07 | <1% |
| Crude death rate and under 5 death rate | Random forest (RF) | Measles incidence (categorical), malaria incidence (categorical), Combined Drought Index (lag 5mths), SAM admissions rate (lag 1mth), Terms of trade (wage to cereal) (lag 4mths) | 2.78e-10 | <1% | 4.32e-09 | <1% |
| Crude death rate and under 5 death rate | Generalised Additive (GAM) | Measles incidence (categorical), malaria incidence (categorical), Combined Drought Index (lag 5mths), SAM admissions rate (lag 1mth) | 1.38e-06 | <1% | 1.39e-06 | <1% |

† Root mean square error. Lower values indicate better predictive performance.

Figure S6. Model performance on leave-one-out cross-validation of A) GLM, B) GLMM, C) GAM and D) RF models of the crude death rate. Each dot is one district-trimester fold.

Based on model comparison using LOOCV, we selected a GLMM with a log link and a Poisson likelihood. The final model includes fixed effects for Composite Drought Index (lagged by 5 months), measles incidence, severe acute malnutrition admission rate (lagged by one month) and malaria incidence, with a random intercept for district.

While bias was minimal across the full dataset, Figure S7 suggests some clustering of under-estimation (year 2018) and specific regions where bias was considerable (e.g. Bay in the south and Woqooyi Galbeed in the north-west).

**Figure S7.** Absolute difference in predicted versus observed deaths in surveyed households, by region (administrative level 1) and year of survey completion.

### Step 5: Estimate excess death toll

We estimated a crude and under 5 years death toll for each district-month in the analysis period by multiplying the estimated population denominator by the number of days in that month and the death rate predicted by the model given the values of predictors for that district-month. To propagate uncertainty, we sampled randomly 1000 times from both the normal distribution of the log death rate and its standard error and the empirical distribution of estimated population denominators.

For each of the 1000 simulated death tolls, we performed aggregation across space or time (e.g. by region or countrywide; by year; overall) as desired and subtracted the total death toll from the counterfactual to obtain excess mortality. The distribution of 1000 random draws were used to define uncertainty intervals, with the 2.5th and 97.5th percentiles reported as the 95% confidence interval.

Table S3 provides excess death toll estimates for different time and geographic strata. Negative excess mortality implies that death rates estimated under observed conditions were lower than the counterfactual level.

Table S4. Estimated excess death tolls by period, age group and region, adopting 2015-2016 death rates as the counterfactual baseline. Figures are rounded to the nearest hundred. Quantities in parentheses are the 95% confidence interval.

| **Region** | **January 2017 to December 2017** | | **January 2018 to December 2018** | | **January 2019 to June 2019** | |
| --- | --- | --- | --- | --- | --- | --- |
|  | All ages | Children under 5y old | All ages | Children under 5y old | All ages | Children under 5y old |
| Awdal | 1,300  (-600 to 2,900) | 300  (-300 to 800) | 3,600  (700 to 5,700) | 2,200  (800 to 3,200) | -700  (-1,400 to 0) | -100  (-400 to 100) |
| Bakool | 1,000  (-100 to 1,700) | 500  (0 to 800) | 2,900  (300 to 4,800) | 1,900  (700 to 2,800) | 300  (-200 to 700) | 100  (-100 to 200) |
| Banadir | 9,800  (8,100 to 11,500) | 8,500 (7,100 to 9,800) | 1,600 (-600 to 4,400) | 1,200  (-200 to 2,100) | 2,500  (1,600 to 3,400) | 2,300  (1,700 to 2,900) |
| Bari | 700  (-100 to 1,500) | 600  (100 to 1,000) | 2,700  (100 to 4,700) | 1,600  (300 to 2,500) | 200  (-200 to 500) | 100  (-100 to 300) |
| Bay | 3,500  (2,100 to 4,700) | 1,800  (1,900 to 3,500) | 2,400  (-300 to 4,400) | 1,400  (200 to 2,300) | 1,500  (800 to 2,100) | 1,000  (500 to 1,300) |
| Galgaduud | 2,000  (400 to 3,100) | 1,100  (300 to 1,700) | 2,000  (-600 to 3,900) | 1,200  (0 to 2,100) | 200  (-400 to 700) | 100  (-200 to 300) |
| Gedo | 1,600  (0 to 2,700) | 1,000  (200 to 1,500) | 2,600  (-200 to 4,600) | 1,400  (100 to 2,300) | 400  (-300 to 1,000) | 200  (-100 to 500) |
| Hiraan | 1,300  (-200 to 2,300) | 900  (200 to 1,400) | 2,600  (-100 to 4,600) | 1,300  (100 to 2,200) | 200  (-400 to 600) | 100  (-200 to 300) |
| Lower Juba | 1,600  (-800 to 3,100) | 1,100  (100 to 1,700) | 1,100  (-1,400 to 2,800) | 600  (-400 to 1,400) | 800  (-400 to 1,500) | 400  (0 to 700) |
| Lower Shabelle | 4,700  (-3,300 to 9,800) | 2,600  (-500 to 4,600) | 1,000  (-1,500 to 2,800) | 500  (-600 to 1,300) | 1,600  (-1,800 to 3,800) | 600  (-600 to 1,400) |
| Middle Juba | 1,400  (-800 to 2,700) | 500  (-200 to 1,000) | 700 (-2,400 to 1,600) | 200  (-800 to 900) | 700  (-400 to 1,400) | 300  (-100 to 600) |
| Middle Shabelle | 1,900  (-1,400 to 4,100) | 1,200  (-400 to 2,200) | 1,500  (-1,900 to 3,900) | 200  (-800 to 1,000) | 500  (-1,000 to 1,500) | 300  (-400 to 700) |
| Mudug | 900 (-300 to 1,700) | 500  (-100 to 900) | 600  (-1,900 to 2,400) | 700  (-400 to 1,500) | 100 (-400 to 500) | 100 (-200 to 300) |
| Nugaal | 400  (0 to 700) | 300  (100 to 600) | 900  (-1,400 to 2,600) | 700  (-300 to 1,400) | -100  (-300 to 100) | 0  (-100 to 100) |
| Sanaag | 2,000  (400 to 3,400) | 700  (100 to 1,200) | 1,500  (-1,100 to 3,400) | 1,000  (-100 to 1,800) | 200  (-500 to 700) | 100  (-200 to 300) |
| Sool | 700  (-200 to 1,300) | 300  (-100 to 600) | 2,500  (-100 to 4,400) | 1,400  (200 to 2,300) | 200  (-200 to 500) | 100  (-100 to 200) |
| Togdheer | 700  (-500 to 1,700) | 300  (-200 to 700) | 1,200  (-1,500 to 3,300) | 600  (-600 to 1,400) | -200  (-700 to 300) | 0  (-300 to 100) |
| Woqooyi Galbeed | 2,500  (1,200 to 3,700) | 500  (0 to 900) | 2,500  (-200 to 4,400) | 1,400  (200 to 2,200) | 700  (100 to 1,300) | 200  (-100 to 400) |
| **Total** | **37,800**  **(4,200 to 62,500)** | **22,700**  **(8,100 to 35,300)** | **33,900**  **(-14,300 to 66,900)** | **19,400**  **(-1,500 to 34,600)** | **9,200**  **(-6,100 to 20,600)** | **5,700**  **(-1,100 to 10,600)** |

## Additional analyses

### Comparison between IPC map and ground mortality data availability

Figure S8 shows the spatial distribution of IPC phases in 2022 alongside the availability of ground survey data for the same year, represented by the number of households sampled per survey cluster. Areas classified in higher IPC phases (3-4), indicating more severe food insecurity, are predominantly located in south-central Somalia. These areas are also characterised by relatively dense survey coverage, as reflected by the presence and size of survey centroids.

Figure S8. Spatial distribution of IPC phases in 2022 and ground mortality data availability, represented by survey cluster sample sizes.

### Comparison between composite crisis index and mortality estimates

In Figure S9, we compare trends in estimated death rates at the country-wide scale with a composite index of crisis-emergent risk factors produced by the Food Security and Nutrition Analysis Unit for Somalia (7). This index (<https://dashboard.fsnau.org/index/about-us>) is the result of a consultative process and combines multiple crisis-relevant indicators covering climate (rainfall, vegetation coverage, river levels and price of water), markets (cereal prices, livestock prices, wage labour and terms of trade), health (measles, acute watery diarrhoea, polio and malaria incidence), nutrition (new admissions to therapeutic feeding) and displacement. The index quantifies the number of indicators that are in the alarm phase according to arbitrarily set thresholds; higher values thus indicate high crisis severity.

The comparison with our findings shows that death rates followed similar trends to those of the crisis index, but peaking and declining with a delay of approximately 3-4 months.

Figure S9. Comparison of monthly trends in a composite crisis index, crude death rate and under 5 years death rate. For readability only point estimates are shown.

### Sensitivity analysis A: Impact of population denominator uncertainty

To assess the robustness of our mortality estimates to uncertainty in population size, we performed a sensitivity analysis by assuming that reconstructed district-month population denominators were downward- or upward-biassed by 20%. Figure S10 indicates that the mortality trends remained stable under either sensitivity assumption, with only modest shifts in absolute levels. This suggests that our estimates are relatively robust to plausible errors in population size.

Figure S10. Estimated countrywide deaths per month according to estimated population and assumptions of 20% over- or underestimation in the population estimates.

### Sensitivity analysis B: Impact of under-detection of under-5 deaths

Survey-based estimates of under 5 years mortality may underestimate true values due to recall and reporting biases (1), particularly when the questionnaire is not implemented rigorously or cultural stigma impedes disclosure of deaths among young children. To test the sensitivity of our results to such potential underestimation, we inflated the observed U5DRs by 25% or 50% by randomly modifying the vital status of individual household members, assuming that the simulated additional deaths would occur at the midpoint of the recall period, and recalculating person-time accordingly. As shown in Figure S11, these assumed levels of under-reporting increased the estimated U5DR levels by ~0.1 to 0.3 deaths per 10,000 child-days on average, but the temporal trends and relative fluctuations over time remained consistent. The main conclusions of our analysis therefore remain valid even if the under-detection of child deaths was significant.

Figure S11. Estimated excess death tolls among all ages and children aged under 5 years for year 2023 based on unadjusted estimates and assumptions of 25% or 50% under-reporting of under 5 years deaths in surveys.

### Trends in survey estimates over time

Figure S12 shows trends in the CDR point estimate of each survey included in the analysis, by typology of community surveyed. As shown, there is no clear evidence of increased point estimates during the first few waves of SARS-CoV-2, whether in urban, IDP or other communities, though some surveys do report relatively high death rates. By contrast, increases are evident during the two drought periods analysed (before and after the pandemic). Note that this crude visualisation does not account for differing survey sample sizes and sampling universes and should not be confused with countrywide mortality trends resulting from model predictions, as shown in the main text.

Figure S12. Trends in crude death rate survey point estimates, by type of community surveyed. Each segment represents the recall period of a given survey. The dotted line is a smoothed local regression trend fitted through the midpoint of survey recall periods. The yellow-shaded area indicates the COVID-19 pandemic period from the date first reported case in Somalia to the end of 2022, comprising > 90% of reported cases.

## References

1.	Checchi F, Testa A, Gimma A, Koum-Besson E, Warsame A. A method for small-area estimation of population mortality in settings affected by crises. Population Health Metrics. 2022 Jan 11;20(1):4. doi:10.1186/s12963-022-00283-6

2.	Ouchtar Y, Ali DA, Ahmed YA, Checchi F. Reconstructing Somalia’s population: A district level analysis. PLOS Global Public Health. 2025 Sep 25;5(9):e0005215. doi:10.1371/journal.pgph.0005215

3.	Buuren S van, Groothuis-Oudshoorn K. mice: Multivariate Imputation by Chained Equations in R. Journal of Statistical Software. 2011 Dec 12;45:1–67. doi:10.18637/jss.v045.i03

4.	Funk C, Peterson P, Landsfeld M, Pedreros D, Verdin J, Shukla S, et al. The climate hazards infrared precipitation with stations—a new environmental record for monitoring extremes. Scientific Data. 2015 Dec 8;2:150066. doi:10.1038/sdata.2015.66 PubMed PMID: 26646728.

5.	Raleigh C, Linke A, Hegre H, Karlsen J. Introducing ACLED: An Armed Conflict Location and Event Dataset. Journal of Peace Research. 2010;47(5):651–60.

6.	The FSNAU Early Warning Early Action Dashboard | FSNAU [Internet]. [cited 2024 Oct 22]. Available from: https://fsnau.org/special-content/fsnau-early-warning-early-action-dashboard

7.	Somalia Early Warning-Early Action Dashboard Time Series Chart: Trends in the Number of Risk Factors in Alarm Phase (Jan 2015 - Jan 2026) - Somalia | ReliefWeb [Internet]. 2026 [cited 2026 Mar 17]. Available from: https://reliefweb.int/report/somalia/somalia-early-warning-early-action-dashboard-time-series-chart-trends-number-risk-factors-alarm-phase-jan-2015-jan-2026
